# Deadly pressure pneumothorax after withdrawal of misplaced feeding tube: a case report

**DOI:** 10.1186/s13256-016-0813-y

**Published:** 2016-02-03

**Authors:** Erik Nygaard Andresen, Martin Frydland, Lotte Usinger

**Affiliations:** 1 Department of Internal Medicine, University Hospital Glostrup, Nordre Ringvej 57, 2600 Glostrup, Denmark; 2 The Heart Centre, Department of Cardiology, Copenhagen University Hospital, Rigshospitalet, Blegdamsvej 9, 2100 Copenhagen, Denmark

**Keywords:** Nasogastric, Tube, Pneumothorax, Tension, Deadly

## Abstract

**Background:**

Many patients have a nasogastric feeding tube inserted during admission; however, misplacement is not uncommon. In this case report we present, to the best of our knowledge, the first documented fatality from pressure pneumothorax following nasogastric tube withdrawal.

**Case presentation:**

An 84-year-old Caucasian woman with dysphagia and at risk of aspiration underwent routine insertion of a nasogastric feeding tube; however, shortly after insertion she developed respiratory distress. A chest X-ray showed the tube had been misplaced into our patient’s right lung. The tube was removed, but our patient died less than an hour after withdrawal. The autopsy report stated that cause of death was tension pneumothorax, which developed following withdrawal of the misplaced feeding tube.

**Conclusions:**

The indications for insertion of nasogastric feeding tubes are many and the procedure is considered harmless; however, if the tube is misplaced there is good reason to be cautious on removal as this can unmask puncture of the pleura eliciting pneumothorax and, as this case report shows, result in an ultimately deadly tension pneumothorax.

## Background

Annually, more than 271,000 nasogastric tubes are inserted in the UK [1]. In patients unable to feed on their own or in need of acute gastric decompression this is a necessary and lifesaving procedure as part of modern medical care.

Although considered a low-risk procedure, insertion of nasogastric tubes involves an alarming rate of misplacements and fatalities. Even though feeding into the lungs is the most common cause of death and serious incidences, examples of pneumothorax and esophageal perforation can easily be found in the literature [1, 2]. One retrospective study [3] from a major tertiary referral university hospital in the US showed that as many as 3.2 % of nasogastric tubes were radiographically documented to be misplaced into the lower airways. A total of 1.2 % of the procedures led to pneumothorax, and 0.5 % involved subsequently lethal complications. The risks inherent in the procedure were found to be the same, regardless of age, gender or in-hospital location (ward versus intensive care unit).

The purpose of this report is to illustrate the potentially serious risks that removal of a misplaced feeding tube poses to patients. Documentation of pneumothorax, and even development of tension pneumothorax after removal, has been described previously [4–6], but this case is, to the best of our knowledge, the first one documented involving fatal tension pneumothorax that developed following removal of a feeding tube.

## Case presentation

An 84-year old Caucasian woman suffering from chronic obstructive pulmonary disease, hypertension, and ulcerative colitis was admitted with suspected atrial fibrillation. During hospitalization she suffered a cerebrovascular accident, resulting in left-sided neurological deficits and dysphagia. A nasogastric tube was inserted routinely due to the risk of aspiration. Despite an uncomplicated placement procedure by an experienced nurse, our patient developed respiratory distress a few hours later.

A physical examination, including normal lung stetoscopia, gave no obvious explanation for the respiratory distress.

**Fig. 1:**
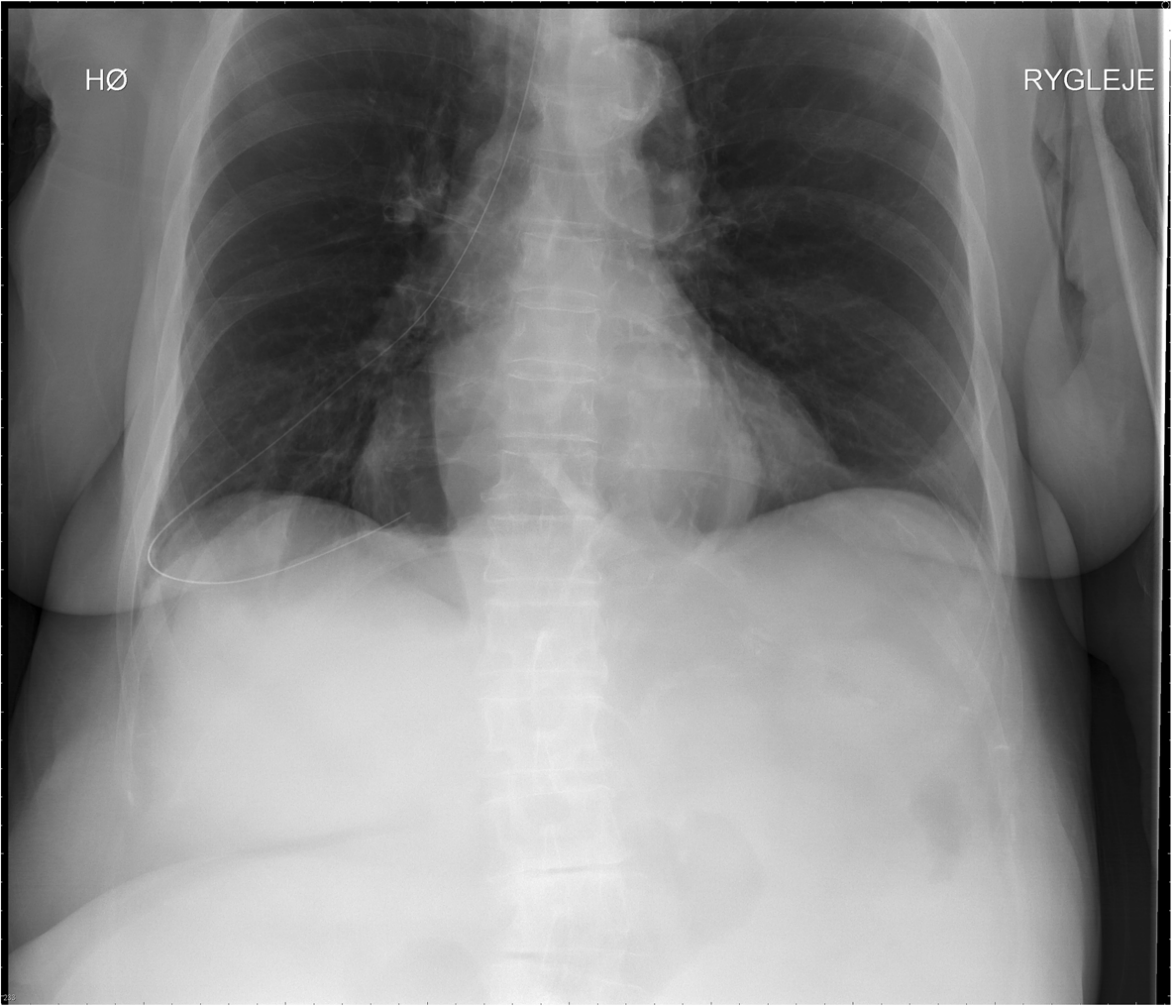
Chest X-ray prior to removal, patient lying down.

A chest X-ray (Fig. 1) revealed the tube had been misplaced into her right lung, descending into the lower lobe, but with no pneumothorax. The tube was easily removed; however, our patient died unexpectedly within the hour. An autopsy revealed a small puncture of the right lower pulmonary lobe and air escaping the thoracic cavity when opened under water. Furthermore the lungs and the mediastinum were found to be displaced into the left thoracic cavity. Cause of death was diagnosed as tension pneumothorax.

Tension pneumothorax was probably caused by removal of the nasogastric tube, since a chest X-ray prior to removal was normal.

## Discussion

We present a multimorbid octogenarian who died from tension pneumothorax, which developed shortly after removal of a misplaced nasogastric feeding tube.

The need for nasogastric tubes in modern medical care is beyond discussion. The methods of ensuring correct positioning of the tube before initiating feeding have been thoroughly described in the literature, and confirmation of pH and the presence of bilirubin are the only proven reliable bedside tests. Chest X-ray is considered the gold standard for determining correct tube position [2]; however, only scant attention is given in the literature to the process of removing a misplaced tube [7]. In agreement with previous case reports [4–6], we consider removing a misplaced nasogastric tube from the lungs a high-risk procedure and one that should be done with great care. Physicians and nurses should consider the risk of pneumothorax when removing a misplaced tube, and the patient should be closely monitored in the hours following removal. Chest X-ray should be considered routine following removal of all tubes known or thought to be misplaced.

## Conclusions

In this case report we document a tension pneumothorax with fatal outcome arising after withdrawal of a misplaced nasogastric feeding tube. To the best of our knowledge, this is the first event of its kind documented. We wish to increase awareness in respect of the dangers a misplaced feeding tube represents, particularly on withdrawal.

## Consent

Written informed consent was obtained from the patient’s next-of-kin for publication of this case report and any accompanying images. A copy of the written consent is available for review by the Editor-in-Chief of this journal.
